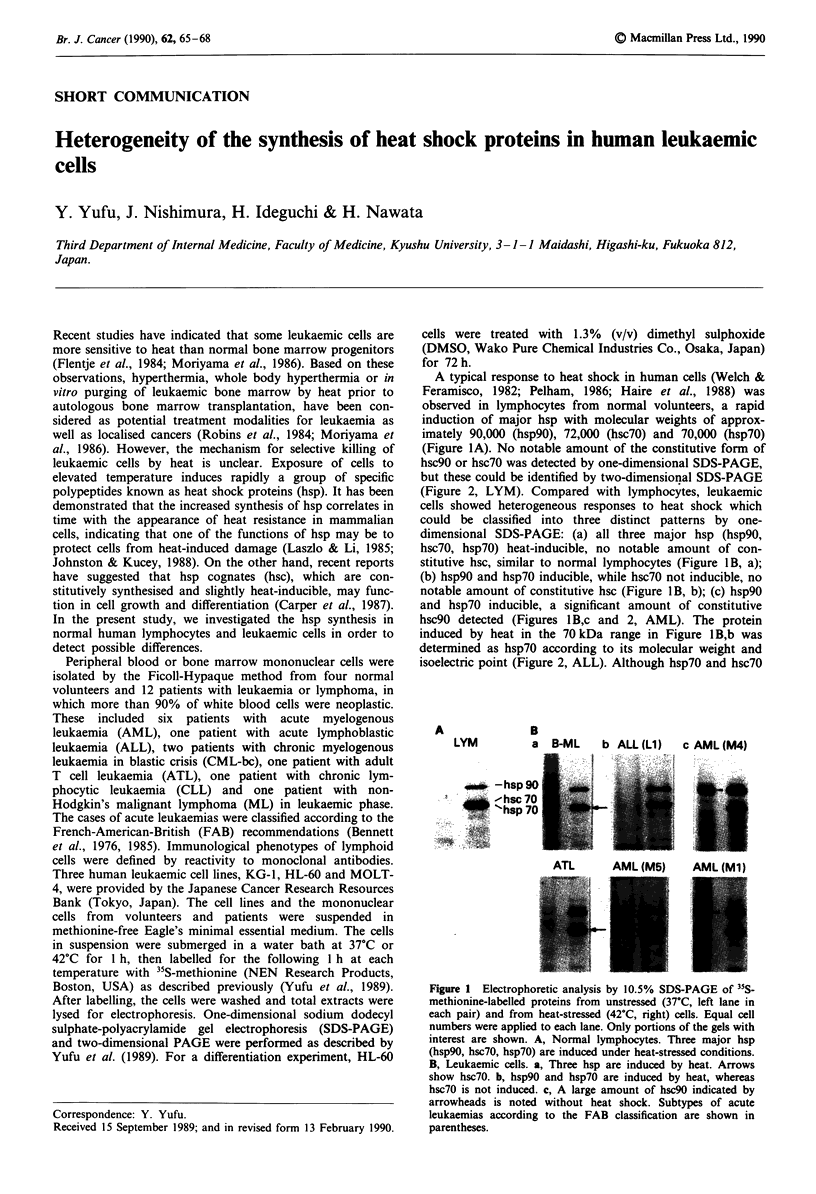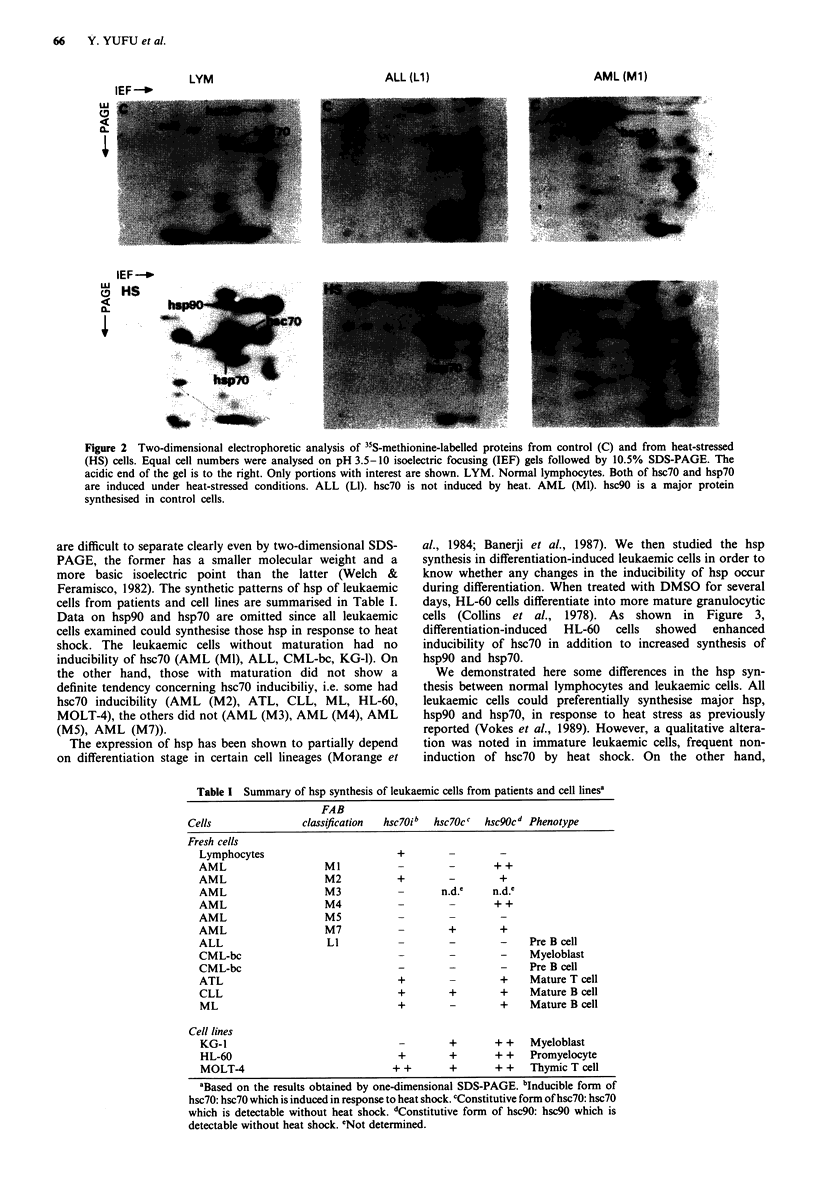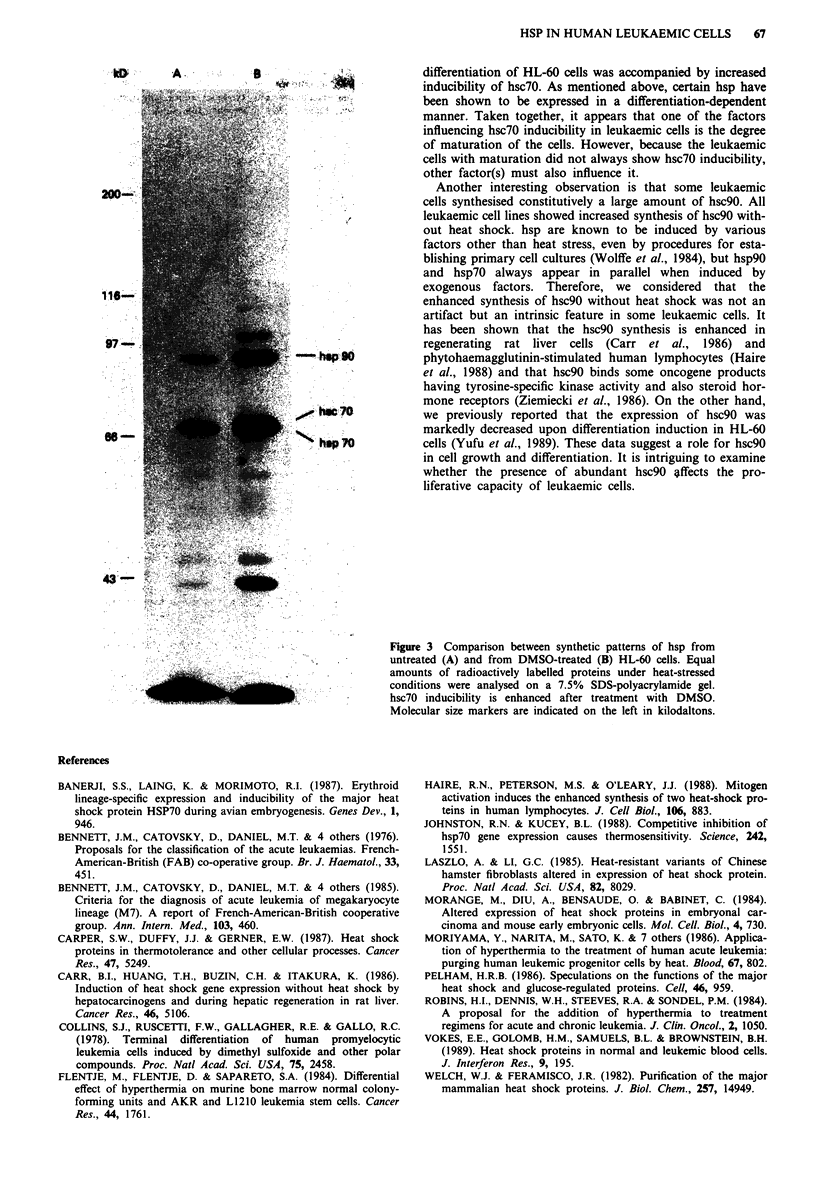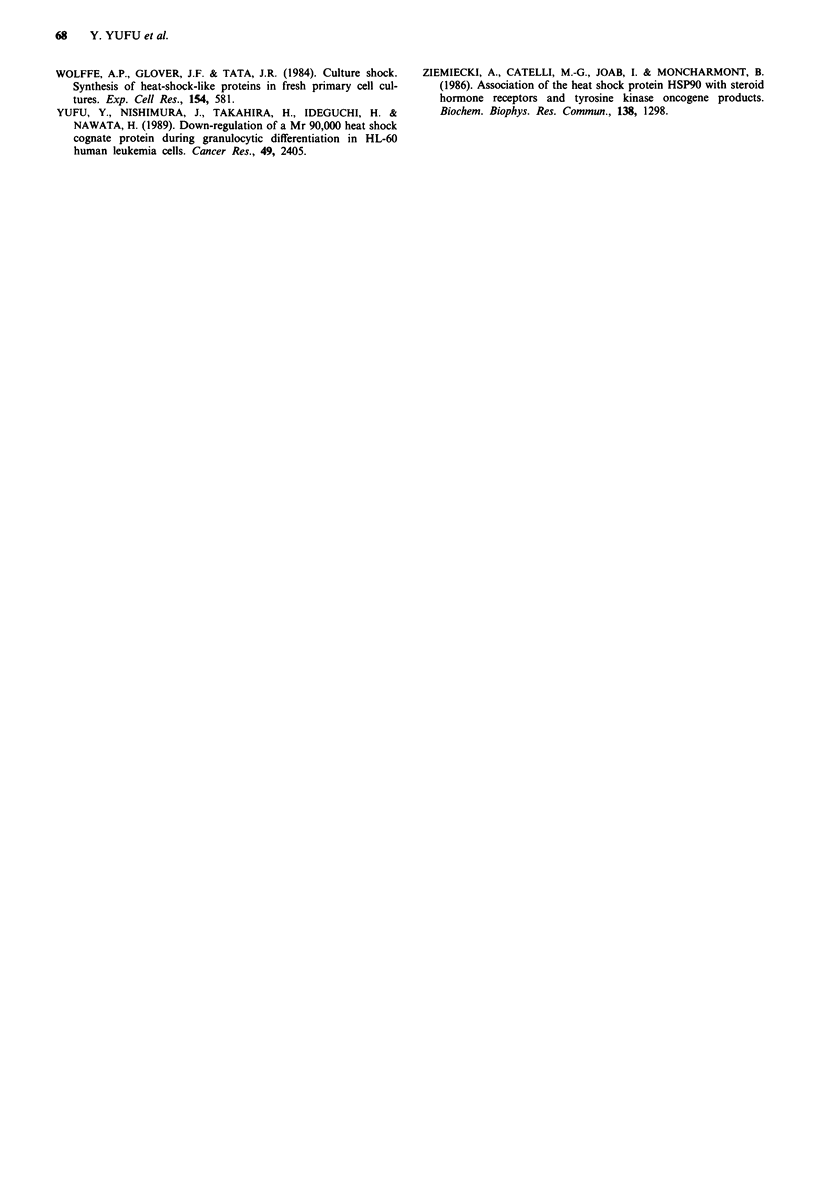# Heterogeneity of the synthesis of heat shock proteins in human leukaemic cells.

**DOI:** 10.1038/bjc.1990.230

**Published:** 1990-07

**Authors:** Y. Yufu, J. Nishimura, H. Ideguchi, H. Nawata

**Affiliations:** Third Department of Internal Medicine, Faculty of Medicine, Kyushu University, Fukuoka, Japan.

## Abstract

**Images:**


*Br. J. Cancer* (1990), **62**, 65–68 © Macmillan Press Ltd., 1990

SHORT COMMUNICATION

### Heterogeneity of the synthesis of heat shock proteins in human leukaemic cells

Y. Yufu, J. Nishimura, H. Ideguchi & H. Nawata

*Third Department of Internal Medicine, Faculty of Medicine, Kyushu University, 3-1-1 Maidashi, Higashi-ku, Fukuoka 812, Japan.*

Recent studies have indicated that some leukaemic cells are more sensitive to heat than normal bone marrow progenitors (Flentje *et al.*, 1984; Moriyama *et al.*, 1986). Based on these observations, hyperthermia, whole body hyperthermia or *in vitro* purging of leukaemic bone marrow by heat prior to autologous bone marrow transplantation, have been considered as potential treatment modalities for leukaemia as well as localised cancers (Robins *et al.*, 1984; Moriyama *et al.*, 1986). However, the mechanism for selective killing of leukaemic cells by heat is unclear. Exposure of cells to elevated temperature induces rapidly a group of specific polypeptides known as heat shock proteins (hsp). It has been demonstrated that the increased synthesis of hsp correlates in time with the appearance of heat resistance in mammalian cells, indicating that one of the functions of hsp may be to protect cells from heat-induced damage (Laszlo & Li, 1985; Johnston & Kucey, 1988). On the other hand, recent reports have suggested that hsp cognates (hsc), which are constitutively synthesised and slightly heat-inducible, may function in cell growth and differentiation (Carper *et al.*, 1987). In the present study, we investigated the hsp synthesis in normal human lymphocytes and leukaemic cells in order to detect possible differences.

Peripheral blood or bone marrow mononuclear cells were isolated by the Ficoll-Hypaque method from four normal volunteers and 12 patients with leukaemia or lymphoma, in which more than 90% of white blood cells were neoplastic. These included six patients with acute myelogenous leukaemia (AML), one patient with acute lymphoblastic leukaemia (ALL), two patients with chronic myelogenous leukaemia in blastic crisis (CML-bc), one patient with adult T cell leukaemia (ATL), one patient with chronic lymphocytic leukaemia (CLL) and one patient with non-Hodgkin's malignant lymphoma (ML) in leukaemic phase. The cases of acute leukaemias were classified according to the French-American-British (FAB) recommendations (Bennett *et al.*, 1976, 1985). Immunological phenotypes of lymphoid cells were defined by reactivity to monoclonal antibodies. Three human leukaemic cell lines, KG-1, HL-60 and MOLT-4, were provided by the Japanese Cancer Research Resources Bank (Tokyo, Japan). The cell lines and the mononuclear cells from volunteers and patients were suspended in methionine-free Eagle's minimal essential medium. The cells in suspension were submerged in a water bath at 37°C or 42°C for 1 h, then labelled for the following 1 h at each temperature with $^{35}$S-methionine (NEN Research Products, Boston, USA) as described previously (Yufu *et al.*, 1989). After labelling, the cells were washed and total extracts were lysed for electrophoresis. One-dimensional sodium dodecyl sulphate-polyacrylamide gel electrophoresis (SDS-PAGE) and two-dimensional PAGE were performed as described by Yufu *et al.* (1989). For a differentiation experiment, HL-60 cells were treated with 1.3% (v/v) dimethyl sulphoxide (DMSO, Wako Pure Chemical Industries Co., Osaka, Japan) for 72 h.

A typical response to heat shock in human cells (Welch & Feramisco, 1982; Pelham, 1986; Haire *et al.*, 1988) was observed in lymphocytes from normal volunteers, a rapid induction of major hsp with molecular weights of approximately 90,000 (hsp90), 72,000 (hsc70) and 70,000 (hsp70) (Figure 1A). No notable amount of the constitutive form of hsc90 or hsc70 was detected by one-dimensional SDS-PAGE, but these could be identified by two-dimensional SDS-PAGE (Figure 2, LYM). Compared with lymphocytes, leukaemic cells showed heterogeneous responses to heat shock which could be classified into three distinct patterns by one-dimensional SDS-PAGE: (a) all three major hsp (hsp90, hsc70, hsp70) heat-inducible, no notable amount of constitutive hsc, similar to normal lymphocytes (Figure 1B, a); (b) hsp90 and hsp70 inducible, while hsc70 not inducible, no notable amount of constitutive hsc (Figure 1B, b); (c) hsp90 and hsp70 inducible, a significant amount of constitutive hsc90 detected (Figures 1B,c and 2, AML). The protein induced by heat in the 70 kDa range in Figure 1B,b was determined as hsp70 according to its molecular weight and isoelectric point (Figure 2, ALL). Although hsp70 and hsc70

**Figure 1** Electrophoretic analysis by 10.5% SDS-PAGE of $^{35}$S-methionine-labelled proteins from unstressed (37°C, left lane in each pair) and from heat-stressed (42°C, right) cells. Equal cell numbers were applied to each lane. Only portions of the gels with interest are shown. **A**, Normal lymphocytes. Three major hsp (hsp90, hsc70, hsp70) are induced under heat-stressed conditions. **B**, Leukaemic cells. **a**, Three hsp are induced by heat. Arrows show hsc70. **b**, hsp90 and hsp70 are induced by heat, whereas hsc70 is not induced. **c**, A large amount of hsc90 indicated by arrowheads is noted without heat shock. Subtypes of acute leukaemias according to the FAB classification are shown in parentheses.

Correspondence: Y. Yufu.
Received 15 September 1989; and in revised form 13 February 1990.

**Figure 2** Two-dimensional electrophoretic analysis of $^{35}S$-methionine-labelled proteins from control (C) and from heat-stressed (HS) cells. Equal cell numbers were analysed on pH 3.5–10 isoelectric focusing (IEF) gels followed by 10.5% SDS-PAGE. The acidic end of the gel is to the right. Only portions with interest are shown. LYM. Normal lymphocytes. Both of hsc70 and hsp70 are induced under heat-stressed conditions. ALL (Ll). hsc70 is not induced by heat. AML (Ml). hsc90 is a major protein synthesised in control cells.

are difficult to separate clearly even by two-dimensional SDS-PAGE, the former has a smaller molecular weight and a more basic isoelectric point than the latter (Welch & Feramisco, 1982). The synthetic patterns of hsp of leukaemic cells from patients and cell lines are summarised in Table I. Data on hsp90 and hsp70 are omitted since all leukaemic cells examined could synthesise those hsp in response to heat shock. The leukaemic cells without maturation had no inducibility of hsc70 (AML (Ml), ALL, CML-bc, KG-l). On the other hand, those with maturation did not show a definite tendency concerning hsc70 inducibiliy, i.e. some had hsc70 inducibility (AML (M2), ATL, CLL, ML, HL-60, MOLT-4), the others did not (AML (M3), AML (M4), AML (M5), AML (M7)).

The expression of hsp has been shown to partially depend on differentiation stage in certain cell lineages (Morange et al., 1984; Banerji et al., 1987). We then studied the hsp synthesis in differentiation-induced leukaemic cells in order to know whether any changes in the inducibility of hsp occur during differentiation. When treated with DMSO for several days, HL-60 cells differentiate into more mature granulocytic cells (Collins et al., 1978). As shown in Figure 3, differentiation-induced HL-60 cells showed enhanced inducibility of hsc70 in addition to increased synthesis of hsp90 and hsp70.

We demonstrated here some differences in the hsp synthesis between normal lymphocytes and leukaemic cells. All leukaemic cells could preferentially synthesise major hsp, hsp90 and hsp70, in response to heat stress as previously reported (Vokes et al., 1989). However, a qualitative alteration was noted in immature leukaemic cells, frequent non-induction of hsc70 by heat shock. On the other hand,

**Table I** Summary of hsp synthesis of leukaemic cells from patients and cell lines[a]

| *Cells* | *FAB classification* | *hsc70i*[b] | *hsc70c*[c] | *hsc90c*[d] | *Phenotype* |
|---|---|---|---|---|---|
| *Fresh cells* | | | | | |
| Lymphocytes | | + | – | – | |
| AML | M1 | – | – | + + | |
| AML | M2 | + | – | + | |
| AML | M3 | – | n.d.[e] | n.d.[e] | |
| AML | M4 | – | – | + + | |
| AML | M5 | – | – | – | |
| AML | M7 | – | + | + | |
| ALL | L1 | – | – | – | Pre B cell |
| CML-bc | | – | – | – | Myeloblast |
| CML-bc | | – | – | – | Pre B cell |
| ATL | | + | – | + | Mature T cell |
| CLL | | + | + | + | Mature B cell |
| ML | | + | – | + | Mature B cell |
| *Cell lines* | | | | | |
| KG-1 | | – | + | + + | Myeloblast |
| HL-60 | | + | + | + + | Promyelocyte |
| MOLT-4 | | + + | + | + + | Thymic T cell |

[a]Based on the results obtained by one-dimensional SDS-PAGE. [b]Inducible form of hsc70: hsc70 which is induced in response to heat shock. [c]Constitutive form of hsc70: hsc70 which is detectable without heat shock. [d]Constitutive form of hsc90: hsc90 which is detectable without heat shock. [e]Not determined.

**Figure 3** Comparison between synthetic patterns of hsp from untreated (A) and from DMSO-treated (B) HL-60 cells. Equal amounts of radioactively labelled proteins under heat-stressed conditions were analysed on a 7.5% SDS-polyacrylamide gel. hsc70 inducibility is enhanced after treatment with DMSO. Molecular size markers are indicated on the left in kilodaltons.

differentiation of HL-60 cells was accompanied by increased inducibility of hsc70. As mentioned above, certain hsp have been shown to be expressed in a differentiation-dependent manner. Taken together, it appears that one of the factors influencing hsc70 inducibility in leukaemic cells is the degree of maturation of the cells. However, because the leukaemic cells with maturation did not always show hsc70 inducibility, other factor(s) must also influence it.

Another interesting observation is that some leukaemic cells synthesised constitutively a large amount of hsc90. All leukaemic cell lines showed increased synthesis of hsc90 without heat shock. hsp are known to be induced by various factors other than heat stress, even by procedures for establishing primary cell cultures (Wolffe *et al.*, 1984), but hsp90 and hsp70 always appear in parallel when induced by exogenous factors. Therefore, we considered that the enhanced synthesis of hsc90 without heat shock was not an artifact but an intrinsic feature in some leukaemic cells. It has been shown that the hsc90 synthesis is enhanced in regenerating rat liver cells (Carr *et al.*, 1986) and phytohaemagglutinin-stimulated human lymphocytes (Haire *et al.*, 1988) and that hsc90 binds some oncogene products having tyrosine-specific kinase activity and also steroid hormone receptors (Ziemiecki *et al.*, 1986). On the other hand, we previously reported that the expression of hsc90 was markedly decreased upon differentiation induction in HL-60 cells (Yufu *et al.*, 1989). These data suggest a role for hsc90 in cell growth and differentiation. It is intriguing to examine whether the presence of abundant hsc90 affects the proliferative capacity of leukaemic cells.

**References**

BANERJI, S.S., LAING, K. & MORIMOTO, R.I. (1987). Erythroid lineage-specific expression and inducibility of the major heat shock protein HSP70 during avian embryogenesis. *Genes Dev.*, **1**, 946.

BENNETT, J.M., CATOVSKY, D., DANIEL, M.T. & 4 others (1976). Proposals for the classification of the acute leukaemias. French-American-British (FAB) co-operative group. *Br. J. Haematol.*, **33**, 451.

BENNETT, J.M., CATOVSKY, D., DANIEL, M.T. & 4 others (1985). Criteria for the diagnosis of acute leukemia of megakaryocyte lineage (M7). A report of French-American-British cooperative group. *Ann. Intern. Med.*, **103**, 460.

CARPER, S.W., DUFFY, J.J. & GERNER, E.W. (1987). Heat shock proteins in thermotolerance and other cellular processes. *Cancer Res.*, **47**, 5249.

CARR, B.I., HUANG, T.H., BUZIN, C.H. & ITAKURA, K. (1986). Induction of heat shock gene expression without heat shock by hepatocarcinogens and during hepatic regeneration in rat liver. *Cancer Res.*, **46**, 5106.

COLLINS, S.J., RUSCETTI, F.W., GALLAGHER, R.E. & GALLO, R.C. (1978). Terminal differentiation of human promyelocytic leukemia cells induced by dimethyl sulfoxide and other polar compounds. *Proc. Natl Acad. Sci. USA*, **75**, 2458.

FLENTJE, M., FLENTJE, D. & SAPARETO, S.A. (1984). Differential effect of hyperthermia on murine bone marrow normal colony-forming units and AKR and L1210 leukemia stem cells. *Cancer Res.*, **44**, 1761.

HAIRE, R.N., PETERSON, M.S. & O'LEARY, J.J. (1988). Mitogen activation induces the enhanced synthesis of two heat-shock proteins in human lymphocytes. *J. Cell Biol.*, **106**, 883.

JOHNSTON, R.N. & KUCEY, B.L. (1988). Competitive inhibition of hsp70 gene expression causes thermosensitivity. *Science*, **242**, 1551.

LASZLO, A. & LI, G.C. (1985). Heat-resistant variants of Chinese hamster fibroblasts altered in expression of heat shock protein. *Proc. Natl Acad. Sci. USA*, **82**, 8029.

MORANGE, M., DIU, A., BENSAUDE, O. & BABINET, C. (1984). Altered expression of heat shock proteins in embryonal carcinoma and mouse early embryonic cells. *Mol. Cell. Biol.*, **4**, 730.

MORIYAMA, Y., NARITA, M., SATO, K. & 7 others (1986). Application of hyperthermia to the treatment of human acute leukemia: purging human leukemic progenitor cells by heat. *Blood*, **67**, 802.

PELHAM, H.R.B. (1986). Speculations on the functions of the major heat shock and glucose-regulated proteins. *Cell*, **46**, 959.

ROBINS, H.I., DENNIS, W.H., STEEVES, R.A. & SONDEL, P.M. (1984). A proposal for the addition of hyperthermia to treatment regimens for acute and chronic leukemia. *J. Clin. Oncol.*, **2**, 1050.

VOKES, E.E., GOLOMB, H.M., SAMUELS, B.L. & BROWNSTEIN, B.H. (1989). Heat shock proteins in normal and leukemic blood cells. *J. Interferon Res.*, **9**, 195.

WELCH, W.J. & FERAMISCO, J.R. (1982). Purification of the major mammalian heat shock proteins. *J. Biol. Chem.*, **257**, 14949.

WOLFFE, A.P., GLOVER, J.F. & TATA, J.R. (1984). Culture shock. Synthesis of heat-shock-like proteins in fresh primary cell cultures. *Exp. Cell Res.*, **154**, 581.

YUFU, Y., NISHIMURA, J., TAKAHIRA, H., IDEGUCHI, H. & NAWATA, H. (1989). Down-regulation of a Mr 90,000 heat shock cognate protein during granulocytic differentiation in HL-60 human leukemia cells. *Cancer Res.*, **49**, 2405.

ZIEMIECKI, A., CATELLI, M.-G., JOAB, I. & MONCHARMONT, B. (1986). Association of the heat shock protein HSP90 with steroid hormone receptors and tyrosine kinase oncogene products. *Biochem. Biophys. Res. Commun.*, **138**, 1298.